# Synthesis, Characterization, and Analysis of Hybrid Carbon Nanotubes by Chemical Vapor Deposition: Application for Aluminum Removal

**DOI:** 10.3390/polym12061305

**Published:** 2020-06-08

**Authors:** Alfarooq O. Basheer, Mohammed Abdulhakim Alsaadi, Wan Zuhairi Wan Yaacob, Y. Al-Douri

**Affiliations:** 1 Department for Earth Sciences and Environment, Faculty of Science and Technology, Universiti Kebangsaan Malaysia, Bangi 43600, Selangor, Malaysia; yaacobzw@ukm.edu.my; 2 Nanotechnology and Catalysis Research Center (NANOCAT), University of Malaya, Kuala Lumpur 50603, Malaysia; m.hakim@unizwa.edu.om (M.A.A.); yarub@um.edu.my (Y.A.-D.); 3 National Chair of Materials Science and Metallurgy, University of Nizwa, Nizawa 611, Oman; 4 Department of Civil Engineering, Almaref University College, Al-Anbar 31001, Iraq; 5 University Research Center, Cihan University Sulaimaniya, Sulaymaniyah 46002, Iraq; 6 Department of Mechatronics Engineering, Faculty of Engineering and Natural Sciences, Bahcesehir University, 34349 Besiktas, Istanbul, Turkey

**Keywords:** synthesis, carbon nanotubes, characterization, chemical vapor deposition, adsorption

## Abstract

Hybrid carbon nanotubes (CNTs) are grown on biomass powder-activated carbon (bio-PAC) by loading iron nanoparticles (Fe) as catalyst templates using chemical vapor deposition (CVD) and using acetylene as carbon source, under specific conditions as reaction temperature, time, and gas ratio that are 550 °C, 47 min, and 1, respectively. Specifications of hybrid CNTs were analyzed and characterized using field emission scanning electron microscope (FESEM) with energy-dispersive X-ray spectroscopy (EDX), transmission electron microscopic (TEM), Fourier-transform infrared (FTIR), X-ray diffraction (XRD), thermogravimetric analysis (TGA), surface area Brunauer–Emmett–Teller (BET), and zeta potential. The results revealed the high quality and unique morphologies of hybrid CNTs. Furthermore, removal and capacity of Al^3+^ were optimized by response surface methodology (RSM). However, the results revealed that the pseudo-second-order model well represented adsorption kinetic data, while the isotherm data were effectively fitted using a Freundlich model. The maximum adsorption capacity was 347.88 mg/g. It could be concluded that synthesized hybrid CNTs are a new cost-effective and promising adsorbent for removing Al^3+^ ion from wastewater.

## 1. Introduction

The environmental quality has been deteriorating on a global scale every day because of the rapid development of industrialization and increasing infrastructure. Industrial effluents, farm wastes, and sewage add major contaminants to the environment as they contain several toxic metals and organic and inorganic pollutants. Toxic metal pollution is caused by the industrial waste discharged in the water ecosystems, which severely threaten humanity and all living creatures on earth. Aluminum is known to be extremely reactive to oxygen and carbon, and a high level of aluminum can harm human health because of its intoxication and result in severe diseases. Hence, it is recognized as the main factor contributing to autism spectrum disorders, Alzheimer’s disease, and neurotoxicity of the central nervous system [1,2,3].

Consequently, drinking water should not contain more than 0.05–0.20 mg/L aluminum concentration, as reported by the Environmental Protection Agency (EPA) [4]. It is thus crucial to remove aluminum before it is discharged into aquatic environments.

To remove toxic metal pollution from wastewater, several conventional methods have been implemented. Those metals can be chemically precipitated with hydroxides, chelating precipitation sulfides, membrane filtration, coagulation and flocculation, bioadsorbents, adsorption by porous carbon materials, electrodialysis, ion exchange, flotation, and electrochemical treatments, such as electrochemical oxidation, electrocoagulation/electrocoagulation flotation, electrodialysis, and photoelectrochemical, sonoelectrochemical, and electrochemical reduction [5,6,7,8,9].

Of those techniques, adsorption is considered to be the superior technique because of its flexibility in design, operation, cost-effectiveness, and enabling large-scale applications. Thus, various types of component of adsorbents have been utilized for removal of toxic metals, such as sawdust [10], rice husks [11], maize cobs [12], granulated blast-furnace slag [13], kaolinitic and giru clay [14], aquatic plant [15], powder-activated carbon (PAC) [16], and nanomaterials [17].

Today, there is increasing interest in nanomaterials because of their excellent mechanical performance. One of the interesting nanomaterials is carbon nanotubes (CNTs), which are been widely studied and evaluated for water treatment because of their exceptional properties, including nanotubes’ structure, easy separation, catalytic potential, high reactivity, small size, and large surface area, which can be effective adsorbents for several metals. CNTs are grown using several methods, such as arc discharge, laser ablation, and chemical vapor deposition (CVD). CVD technique is more effective as it is a cost-effective technique and provides high-purity material under controlled growth conditions [18].

As PAC enjoys wide precursor accessibility with its low cost and chemical alterations potentials, it is considered to be the ideal substrate for CVD. Typically, selective growth is easy to achieve by implementing a synthesis technique that used pre-patterned catalyst nanoparticles, including iron, nickel, molybdenum, aluminum, cobalt, and zirconium. Xiang et al. [19] and Mustafa et al. [20] reported synthesized CNTs on PAC using CVD in which the acetylene was utilized as the source of carbon along with several catalysts obtained from cobalt, iron, aluminum, and nickel. While Haiyam et al. synthesized carbon nanomaterials (CNMs) on commercial PAC loaded with nickel nanoparticles by using methane as a carbon source [21]. Arash et al. synthesized CNTs using CVD on silicon sheets loaded with iron and using molybdenum methane utilized as a carbon source [22]. Moreover, Zaho et al. synthesized a significant outcome concerning the CNTs by using CVD in water and implementing iron and molybdenum catalyst supported by a magnesium oxide substrate [23]. There was a reversal relationship between catalyst lifetime and CNT growth rate, as noted by Chen et al. [24]. Finally, Mamtm et al. reported that PAC could be a good precursor to CNT’s growth [25]. It is believed that PAC’s dose does not require any chemical or physical elimination from the functional bulk material, contrary to other substrates. Hybrid CNTs can be effectively defined as combinations of two or more materials, or of materials and space, assembled in such a way as to have attributes not offered by any one material alone. Furthermore, it was found that iron nanoparticles as catalyst and acetylene as carbon source could provide pure CNTs of high quality and density. The feature of this study was to shed some light on the unique hybrid CNTs growth and their magnetic properties, making it easier to separate it from the liquid.

The present study investigated the impact of synthesized hybrid CNTs on biomass (bio)-PAC as biomass precursor was loaded by iron nanoparticles (Fe) followed by CVD. The morphology and structure properties of hybrid CNTs were characterized and analyzed by energy-dispersive X-ray spectroscopy (EDX), field-emission scanning electron microscopy (FESEM), transmission electron microscopy (TEM), X-ray diffraction (XRD), Fourier-transform infrared spectroscopy (FTIR), thermogravimetric analysis (TGA), Brunauer–Emmett–Teller (BET), and zeta potential. The results showed that hybrid CNTs could easily separate from water because of hydrophobicity. This could provide a scalable process for industrial applications because of its facile operation and high removal performance and would be used as CNTs’ new cost-effectiveness adsorbent. In addition, response surface methodology (RSM) with central composite design (CCD) was used for optimizing Al^3+^ removal. Moreover, adsorption models’ kinetic and isotherms were also examined and discussed.

## 2. Experimental

### 2.1. Materials and Reagents

For this study, iron (III) nitrate nonahydrate Fe(NO_3_)_3_·9H_2_O and acetone were purchased from Friendemann Schemicit (Selangor, Malaysia); aluminum standard solution Al(NO_3_)_3_·9H_2_O, hydrochloric acid (HCl), and sodium hydroxide (NaOH) were obtained from Merck (Selangor, Malaysia); and C_2_H_2_, H_2_, and N_2_ used for hybrid CNTs growth were purchased from Alpha Gas Solution (AGS) (Shah alam, Malaysia). The biomass powder-activated carbon (bio-PAC), which was fabricated and optimized by our group, was utilized for the rapid and cost-effective synthesis of hybrid CNTs [1].

### 2.2. Synthesis of Hybrid CNTs

#### 2.2.1. Catalyst Impregnation

The Fe was utilized as a catalyst and added in 5 mL acetone, then mixed with (2 g) bio-PAC. However, the mixture was sonicated at 60 °C for 99 min until the evaporation of acetone. Subsequently, the bio-PAC/Fe sample was dried at 105 °C for 24 h. Then, the bio-PAC/Fe was calcinated at 400 °C for 2 h under inert gas (purified N_2_, 200 mL/min) [26].

#### 2.2.2. CVD Growth

**Figure 1:**
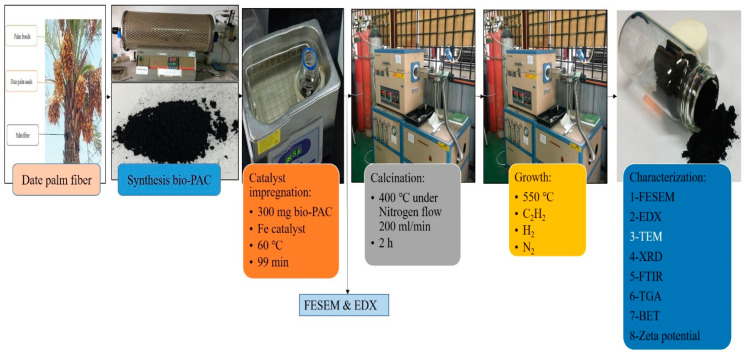
The preparation process for the synthesis of carbon nanotubes (CNTs).

CNTs’ growth was carried out by placing (300 mg) bio-PAC/Fe in a ceramic boat with a CVD reaction tube. A typical growth was accomplished by reduction under H_2_ at 550 °C with the flow (160 mL/min). Thereafter, C_2_H_2_ was used as a carbon source and mixed with H_2_ at a 1:4 ratio. The reaction was passed through a heated reactor for 47 min. After the completion of the reaction, the CVD reactor was cooled to room temperature under purified N_2_ flow rate (200 mL/min), and then the CNTs growth sample was obtained. Figure 1 illustrates the preparation process of hybrid CNTs.

### 2.3. Characterizations

For hybrid CNTs, the surface morphology was analyzed and characterized using field emission scanning electron microscope (FESEM) with energy-dispersive X-ray spectroscopy (EDX), model ZEISS (Merlin, Cambridge, UK), and transmission electron microscope (TEM), (Hitachi-HT7700, Hitachinaka, Japan). The Fourier-transform infrared (FTIR) was utilized in order to analyze both surface functional groups and chemical bonds (Perkin Elmer, San Francisco, CA, USA). The structural phase was analyzed, relying on the powder X-ray diffraction (XRD) by a Burker AXS D8 advance (Karlsruhe, Germany). Whereas the determination of thermal oxidation was obtained from the thermogravimetric analysis (TGA) using the STA-6000 thermal analyzer (Perkin Elmer, Waltham, MA, USA). Moreover, pore size and surface area were calculated by the Brunauer–Emmett–Teller (BET) method (TriStar II 3020, Micromeritics, Norcross, GE, USA). Lastly, the zeta potential was utilized for the measurement of surface charge (Zeta Sizer, Worcestershire, UK).

### 2.4. Adsorption Study

#### 2.4.1. Experimental Design for Optimization of Al^3+^ Adsorption

RSM method was utilized to optimize the removal of Al^3+^. The current study comprehensively examined the three parameters’ impact and interaction, especially on the adsorption of hybrid CNTs’ dosage of 5–20 mg, pH of 3–11, and contact time of 10–120 min. Table 1 displays the actual parameters of each run using the design of expert software (DoE, Stat-Ease, Minneapolis, MN, USA). For this optimization study, an initial aluminum concentration of 5 mg/L was used while agitating the flasks in a shaker at 180 rpm [27].

#### 2.4.2. Kinetic and Isotherm Adsorption

The kinetic study specifies the crucial features of the ions transfer rate, from the solution to the surface of adsorbents, and its associated aspects. To determine the potential adsorbent application, the adsorption system kinetic is relied on to specify the adsorbent competence [28]. Establishing the adsorbent dosage and the pH parameters provides the kinetic study. Meanwhile, the Al^3+^ ion concentrations varied in their values (3 and 5 mg/L) when the kinetic behavior was being studied, and they were managed at various contact times depending on the equilibrium state that was reached at 92 min. This study applied three known kinetic models: the pseudo-first-order, pseudo-second-order, and the intraparticle diffusion model.

The optimum condition of pH, amount of adsorbent dosage, and contact time were also taken into consideration when conducting the isotherm study along with the optimization study. The Freundlich and Langmuir isotherm models are the most known in the isotherm study, with both models frequently describing the Al^3+^ ions adsorption to the CNTs surface where the primary concentration of Al^3+^ varies from 3 to 40 mg/L.

## 3. Results and Discussion

### 3.1. Characterization and Analysis

#### 3.1.1. FESEM, EDX, and TEM of Hybrid CNTs

**Figure 2:**
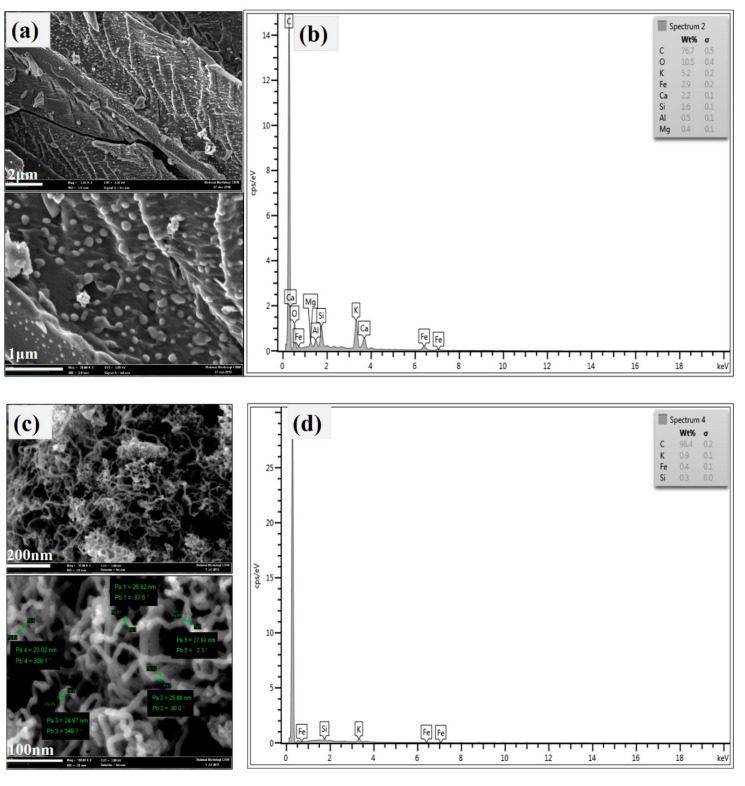
(**a**) FESEM (field emission scanning electron microscope) images; (**b**) EDX (energy-dispersive X-ray spectroscopy) profile of bio-PAC (biomass powder-activated carbon) loaded with Fe catalyst before growth, while (**c**) FESEM images and (**d**) EDX profile for hybrid CNTs after growth.

The morphologies of the hybrid CNTs were obtained using FESEM, as shown in Figure 2. Figure 2a shows that bio-PAC/Fe had a rough surface before growth and that Fe catalyst was dispersed. After C_2_H_2_ decomposition, the hybrid CNTs significantly showed network and agglomeration of CNTs grown on bio-PAC, providing robust evidence of Fe catalyst in the CNTs’ growth, as shown in Figure 2c. Interestingly, the hybrid CNTs’ diameter was between 23.02–27.93 nm. Moreover, the proposed reason for the unique CNTs growth was the Fe bottom, and the entire Fe particle pushed off the bio-PAC, indicating a “base or root” growth model. This was caused by the weak interaction between Fe and PAC as the Fe catalyst was physically adsorbed on PAC [29,30].

Moreover, EDX analysis was used to determine elements of materials before and after growth. Figure 2b,d shows EDX profile for bio-PAC/Fe and hybrid CNTs, respectively. Figure 2b shows that the Fe catalyst was successfully impregnated on bio-PAC. Consequently, Fe catalyst contributed to 2.9%, and other impurities were also present, including C at 76.7%, O at 10.5%, and Si, Ca, Al, and Mg at <3%. Furthermore, hybrid CNTs were observed and successfully grown on the bio-PAC surface, with 98.4% of the surface containing carbon. On the other hand, other elements dissipated from the sample, as shown in Figure 2d, because of the reaction process.

**Figure 3:**
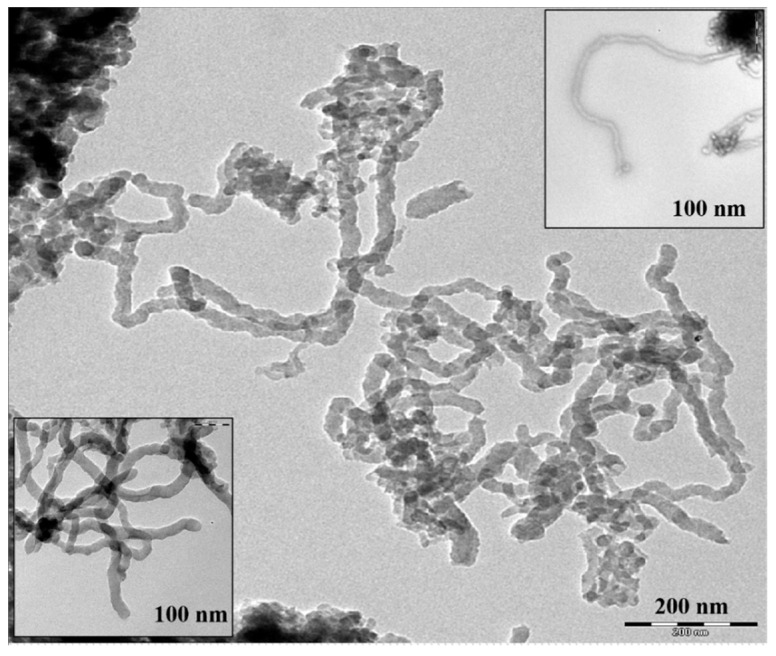
TEM (transmission electron microscope) images for hybrid CNTs.

Furthermore, for more investigation and confirming the carbon nanotubes’ growth, TEM analysis was conducted, as shown in Figure 3. TEM images revealed the internal structure of hybrid CNTs on the bio-PAC surface, which indicated a good quality of CNTs. Thus, the dark spots in the images represented the metallic catalyst clusters where the carbon atoms built up the CNTs’ surrounding structure.

#### 3.1.2. X-ray Diffraction

**Figure 4:**
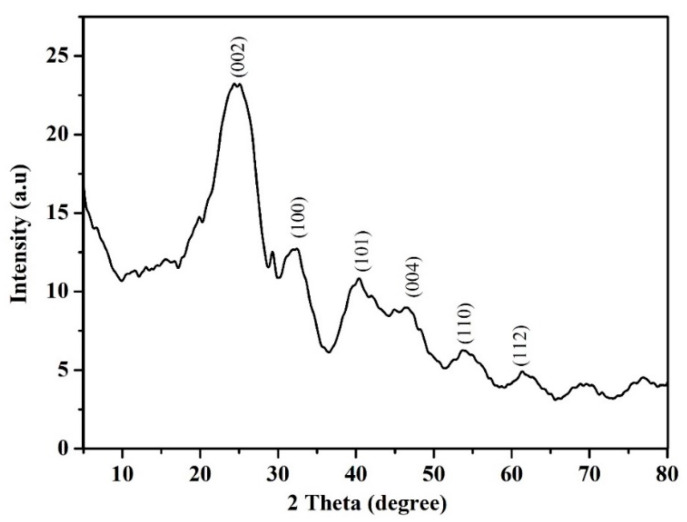
XRD (X-ray diffraction) pattern for hybrid CNTs.

XRD is a rapid investigation method, mainly, which is utilized for the identification of a crystalline and molecular structure material and can provide information on unit cell dimensions [31,32]. Figure 4 shows the XRD pattern of hybrid CNTs; there was the strongest diffraction peak at 2ϴ = 24.31 corresponding to (002) reflection, which corresponded to the crystalline and cylinder structure [33]. However, there were the peaks at 2ϴ, 32.34°, 40.57°, 47.53°, 54.33°, and 62.77° corresponding to (100), (101), (004), (110), and (112) orientation, respectively. All peaks were well matched with the hexagonal graphite structure [34].

#### 3.1.3. Fourier-Transform Infrared (FTIR) Analysis

**Figure 5:**
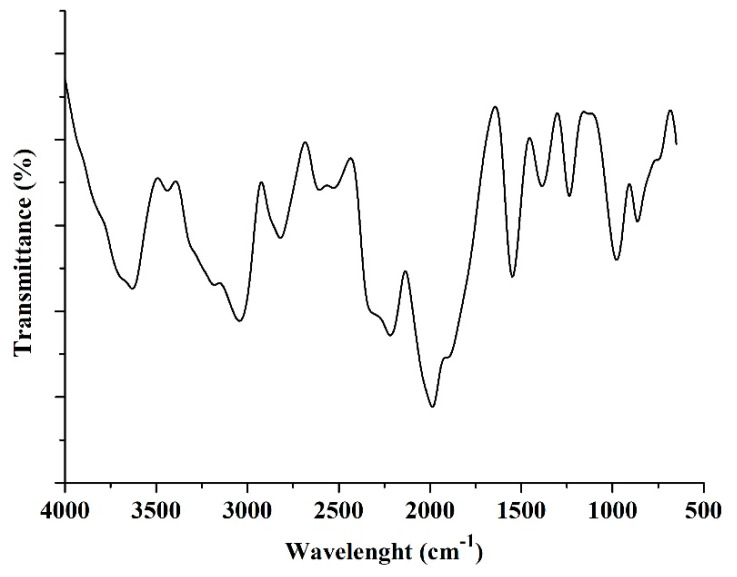
FTIR (Fourier-transform infrared) spectra of hybrid CNTs.

FTIR spectroscopy was used to determine the surface chemistry, i.e., characteristic functional groups of hybrid CNTs, as depicted in Figure 5. As seen in this figure, the weak peaks 3619 cm^−1^ and 3444 cm^−1^ were attributed to O−H stretching vibration, indicating the hydroxyl group on hybrid CNTs surface [35]. The two broad peaks at 2897 and 2819 cm^−1^ could be assigned to C−H stretch vibration. Moreover, the sharp peaks at 2345 and 2227 cm^−1^ indicated C≡C stretching vibrations, and the same results were reported by Minakshi et al. [36,37]. A sharp peak at 1685 cm^−1^ might be attributed to C=C with C=O conjugation or the interaction of the skeletal hybrid CNTs and carboxyl or ketone groups [38,39]. A strong and sharp peak located at 1555 cm^−1^ was attributed to the C=O starching mode of the functional groups on the hybrid CNTs surface, originating from the hybridized carbon [40]. Obviously, peaks at 1392 and 1241 cm^−1^ might also be attributed to N−N and CH−CH_3_ bonds from the intercalated N atoms between the graphite layers of nanotube walls. The band at 1127 cm^−1^ indicated the C−O stretching vibrations in alcohols, phenols, or ether or ester groups [41]. A sharp peak at 867 cm^−1^ was assigned to CH_3_ group vibration.

#### 3.1.4. Thermogravimetric Analysis (TGA)

**Figure 6:**
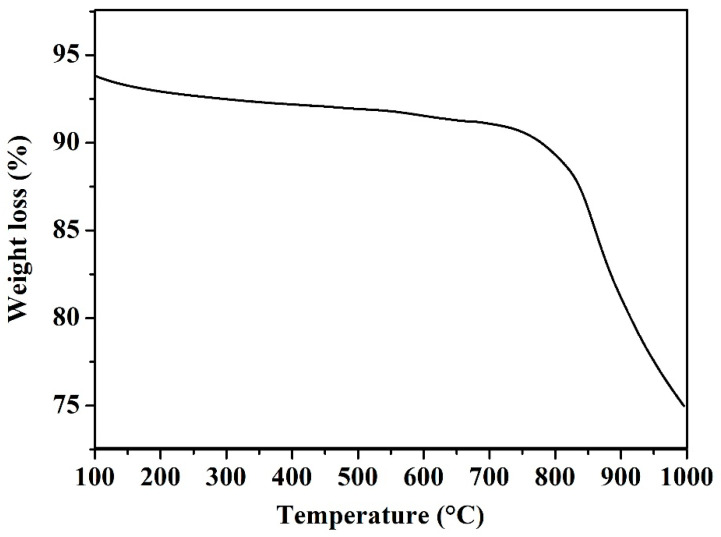
TGA (thermogravimetric analysis) of hybrid CNTs.

TGA was employed to measure the thermal stability of hybrid CNTs. TGA curve, presented in Figure 6, shows a small weight loss resulting from the water removal at approximately 200 °C. The elimination of carbon, along with the oxidation of nanotubes, which occurred at 650–750 °C, was the reason behind the prevailing weight loss steps. However, the weight loss was 18.75% in the temperature range from 100 to 1000 °C, indicating the decomposition of formed carbonaceous material. The results were confirmed with FTIR spectra, as previously illustrated in Figure 4. Moreover, the activation energy of hybrid CNTs oxidation depended on several factors, such as the number of walls, defects, and presence of impurities. Similarly, Misra et al. recounted certain parallel trends concerning the thermogravimetric profiles [42]. Furthermore, when the temperature raised above 1000 °C, the hybrid CNTs were believed to be thermally stable as there was no further decomposition detected.

#### 3.1.5. Brunauer–Emmett–Teller (BET)

**Figure 7:**
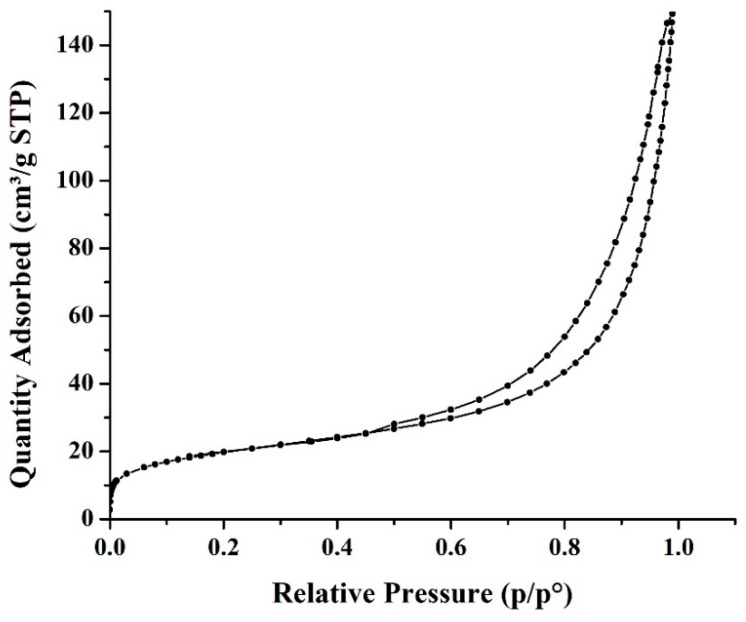
N_2_ adsorption-desorption isotherms.

N_2_ adsorption-desorption isotherms of hybrid CNTs are shown in Figure 7. According to the classification by IUPAC, A-type IV isotherm was observed, which demonstrates the generation of mesopores resulting from hybridization and hysteresis loop corresponding to type H3 [43]. This phenomenon was observed for hybrid CNTs prepared using other substrates [44]. Grown hybrid CNTs decreased in the surface area of 71.42 m^2^/g and pore-volume 0.230 m^3^/g than bio-PAC because of some pores impregnated with Fe nanoparticles (catalyst) for CNTs’ growth. The pore size indicated that mesopores, which were 12 nm, originated from the void spaces between nanotubes and were dominant. Table 2 shows the comparison between surface area, pore-volume, and pore size of different synthesis nanomaterials.

#### 3.1.6. Zeta Potential

It is crucial to classify the surface behavior’s features in the provided aqueous solutions along with the assessment of the suspension stability [49]. Thus, the zeta potential was used to measure the electrical potential on hybrid CNTs’ surface. The numerous pH levels determined the various samples’ zeta potential and their differences. As 10 mg of hybrid CNTs in 20 mL were dispersed, the results observed that zeta potential values varied from 0.111 to −23 (mV) in the pH range of 3–11, as shown in Table 3. The surface charge of hybrid CNTs is positive in acidic medium (pH < pHpzc) [50]. There was also a noticeable reduction in surface charge, which was more negative when pH > pHpzc, causing easy interaction with the positive ions and hybrid CNTs’ surface, and thus, finally affecting the high adsorption process caused by electrostatic interaction [51].

### 3.2. Application Studies

#### 3.2.1. Optimization Study

This study considered 17 experimental runs to evaluate optimum adsorption factors by applying RSM. Thus, the CCD model was used to conduct the experiment. The interaction effect of factors (hybrid CNTs dosage, pH, and contact time) was investigated by fixing 5 mg/L Al^3+^ concentration. Adsorption processes (removal % and capacity mg/g) were adopted as responses, as shown in Table 1. The highest removal was 84% under adsorbent dosage of 13.5 mg/g, pH of 7, and contact time of 22.22 min. However, the highest capacity was 19.95 mg/g at an adsorbent dosage of 5.50 mg/g, pH of 4, and contact time of 18.56 min. The experimental and predicted values were noted to have a close correlation (R2) of Al^3+^ removal and capacity to be 0.9367 and 0.9779, respectively.

The analysis of variance (ANOVA) outcomes for two responses is presented in Table 4 and Table 5, indicating models F-values that were 3.99 and 14.14 in removal and capacity, respectively. This shows that both models are considerable. In this study, the equations of removal (%) were transferred to inverse sqrt (y = 1/√(y + k)) and applied according to Equation (1). Furthermore, the capacity of the significant model was quadratic, which was measured according to Equation (2):

(1) $$\begin{array}{l} 1.0/Sqrt\;\left(Removal\right)\\ \;=+0.10+0.029A-0.086B-0.031C+0.033AB-7.407E\\ \;-003AC+0.11BC+9.708E-003A^{2}+0.12B^{2}+0.016C^{2} \end{array}$$

(2) $$\begin{array}{l} Capacity=+37.10-24.03\;A+20.46\;B+1.03C-21.33AB-1.17AC-6.05BC\\ \;+21.00A^{2}-18.34B^{2}-6.45C^{2} \end{array}$$

where *A* is Dosage, *B* is pH, and *C* is contact time.

**Figure 8:**
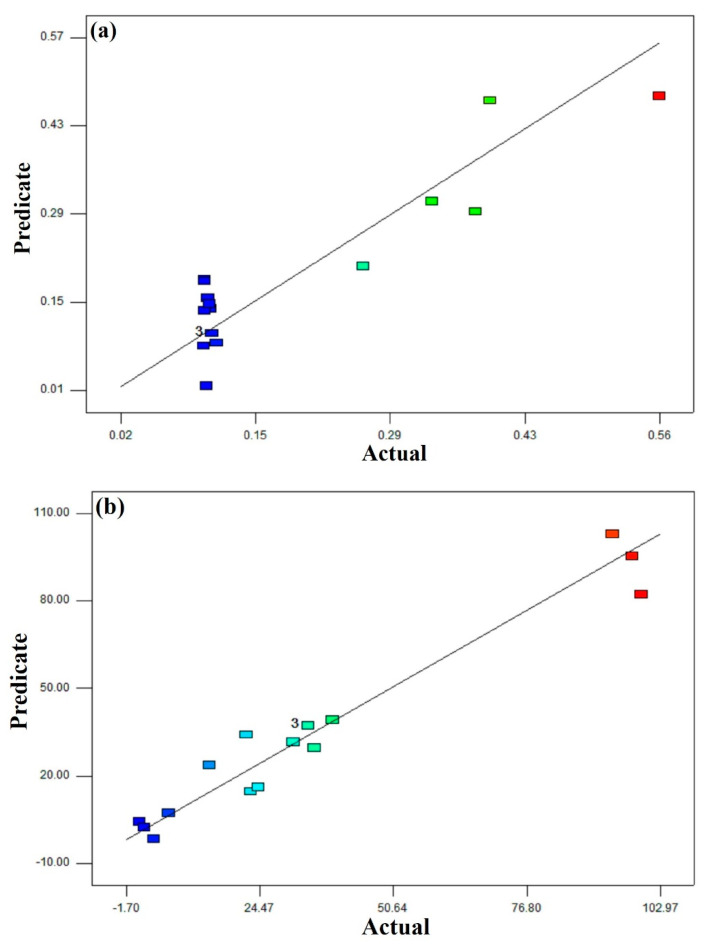
Parity plot of actual and predicate values of (**a**) removal (%) and (**b**) capacity (mg/g).

The comparison between theoretical and experimental values is revealed in Figure 8 for removal (%) and capacity (mg/g). Meanwhile, both the experimental and the theoretical values that were expected by the models established in this research were found to be very close. Therefore, we came to the conclusion that both models had effectively accomplished the interconnection between the process variables.

#### 3.2.2. Effect of Factors on Optimization of Adsorption of Al^3+^ by Hybrid CNTs

**Figure 9:**
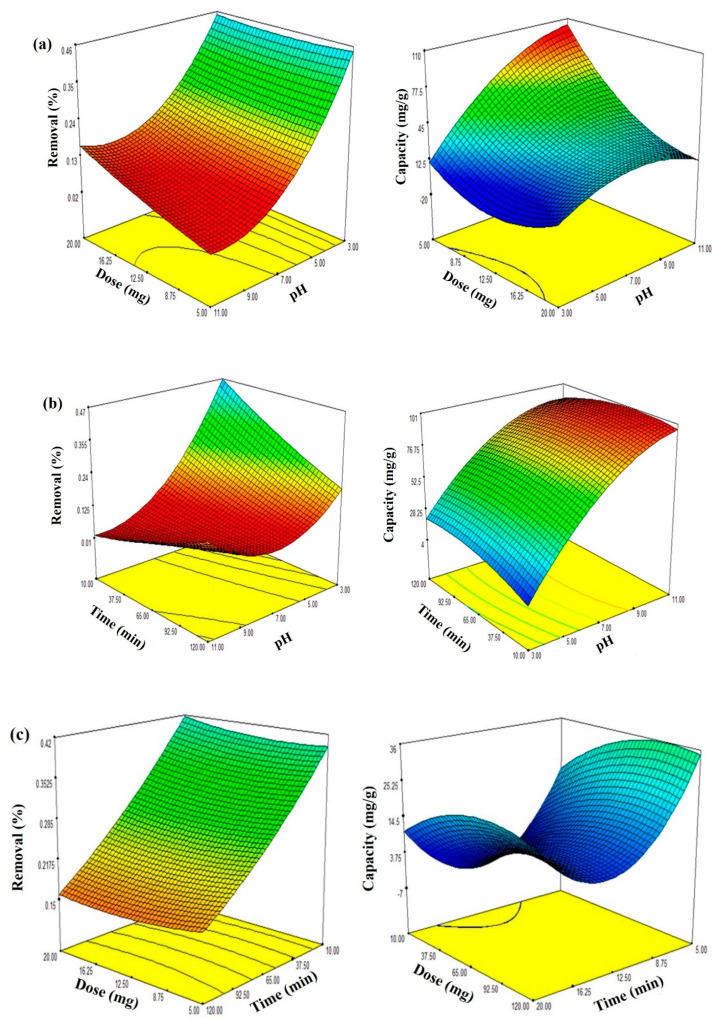
Surface response plots for the effects of (**a**) pH and hybrid CNTs dose, (**b**) pH and contact time, and (**c**) hybrid CNTs dose and contact time.

The section examines the interaction effect of adsorbent dosage and pH on adsorption processes considering removal % and capacity mg/g. Figure 9 illustrates the three-dimensional surface plot, indicating that the effect was more significant between adsorbent dosage and pH on adsorption processes. Thus, it might be related to the increase in the adsorbent amount, which in return increased the active sites’ concentration. As a result, the adsorption solution electrostatic charge was regulated in accordance with the desired adsorption level by eliminating the competitive H^+^ cations. The adsorbent dosage directly increased with removal when the pH and contact time were established. In the meantime, when the adsorbent dosage increased, the capacity declined. This might be associated with the adsorption dosage’s increase and the active adsorption sites’ conglomeration or overlapping [52,53].

In contrast, the interaction impact of contact time and pH on adsorption processes is shown in Figure 9. The system’s failure to reach equilibrium led to a direct correlation of time with removal (%) and capacity (mg/g). Removal (%) increased with an increase of pH to 7, and then it decreased as pH increased. Similarly, the capacity (mg/g) was detected to reach its maximum at a pH of 4, after which it became steady [54].

Toxic metals are well-known precipitators when pH values are high [55]. Therefore, a section of Al in the solution is precipitated in the form of Al^3+^ (NO_3_) because of the impact of OH^−^ anions that are found in the solution [56]. Thus, the initial concentration measurement was taken after adjusting the pH. Consequently, there would be minimum precipitation effects on capacity and removal of hybrid CNTs. Without considering the precipitation phenomena, the overall number of H^+^ cations decreased because of the increase in pH, which was believed to be competing with the Al^3+^ cations of occupying the active sites on the adsorbent. Furthermore, the surface charge of adsorbents could be enhanced when the pH is high [47].

#### 3.2.3. Adsorption Studies

##### Kinetics Studies

**Figure 10:**
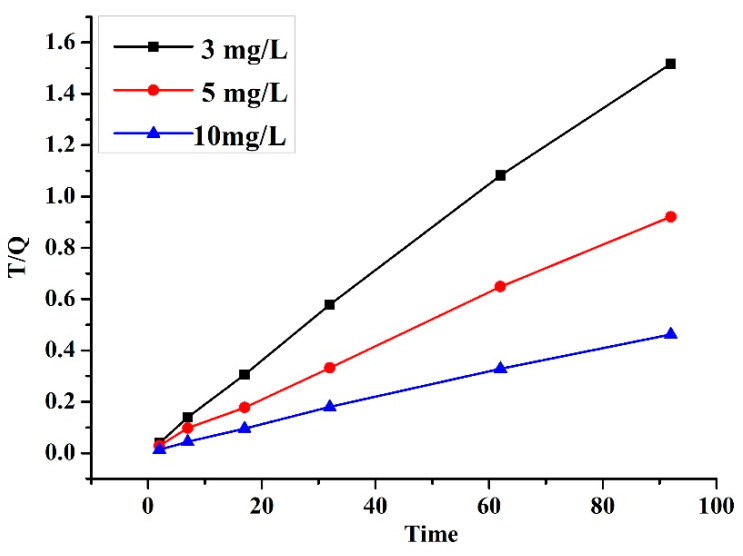
The plot of pseudo-second-order kinetic for Al^3+^ adsorption on hybrid CNTs.

The hybrid CNTs’ adsorbent reaction behavior could be examined through kinetic behavior. For this experimental data, three kinetic models were used, including pseudo-first-order, pseudo-second-order, and the intraparticle diffusion model [57,58]. To conduct the kinetic study, the Al^3+^ that was included varied in concentrations from 3, 5, and 10 mg/L, while the absorption dosage value was fixed at a 13.5 mg and pH of 7 at various time intervals awaiting for the equilibrium state to be attained. According to the three models, it was revealed that the pseudo-second-order fitted better at various Al^3+^ concentrations, which was expressed by a higher correlation coefficient R2. Figure 10 displays the outcomes of the pseudo-second-order. Furthermore, the three applied models for kinetic results are given in Table 6.

##### Isotherm Studies

**Figure 11:**
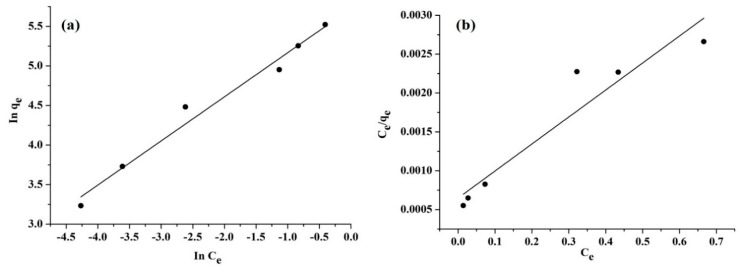
Plots of isotherm models (**a**) Freundlich and (**b**) Langmuir.

The isotherm study was used to perform the Al^3+^ ion adsorption into the hybrid CNTs adsorbent surface through both the Freundlich and Langmuir isotherm models. To conduct the adsorption isotherm study, Al^3+^ was used at multiple concentrations of 3, 5, 10, 20, 30, and 40 mg/L. Moreover, the pH was of 7, while the hybrid CNTs’ dosage was 13.5 mg, and these values were indicated by an optimization study [59]. Figure 11 presents the Freundlich and Langmuir isotherm outcomes. Based on these results, it was discovered that Freundlich was a more suitable fit for Al^3+^ adsorption into hybrid CNTs’ adsorbent surface. The coefficient of correlation (R2) attained from the Freundlich model was 0.9802, while the R2 from the Langmuir model was 0.9738, which indicated that the adsorption of Al^3+^ occurred on the heterogeneous surface with an interaction between the molecules of the adsorbent. Furthermore, Table 7 illustrates a comparison between the maximum adsorption capacities of hybrid CNTs with other nano absorbents materials.

## 4. Conclusions

This study demonstrated that hybrid CNTs could be successfully grown on bio-PAC impregnated with Fe catalyst (bio-PAC/Fe) using acetylene as carbon source and implementing the CVD method under 550 °C for 47 min. The synthesized hybrid CNTs were characterized by EDX, FESEM, and TEM analyses, which confirmed the growth. Thus, results were arranged with FTIR, XRD, BET, TGA, and zeta potential, all of which revealed graphitic structure formation of CNTs that was suggested to be a good adsorbent. Optimum parameters for the Al^3+^ adsorption process were utilized by RSM, and it was found that the hybrid CNTs’ dose was 13.5 mg/g, pH of 7, and contact time of 22.22 min. The adsorption kinetics and isotherms models revealed that the adsorption process was best fitted in the pseudo-second-order and Freundlich model, respectively. Moreover, the maximum adsorption capacity was 347.88 mg/g. Thus, the results revealed that the quality structure of hybrid CNTs was attributed to the supported catalyst (Fe) on the bio-PAC substrate. Moreover, the hybrid CNTs on bio-PAC/Fe, which was originally synthesized from biomass, had a cost-effective adsorbent quality of having the potential to be expanded to other pollutants in wastewater and other applications.

## Figures and Tables

**Table 1:** List of design of experiments runs and the actual values obtained from each response.

Run	Dosage (mg/g)	pH	Time (min)	Response Removal (%)	Response Capacity (mg/g)
1	5	11	10	93.62	93.62
2	20	3	120	96.74	24.185
3	12.50	7	65	84.68	33.872
4	12.50	7	65	84.68	33.872
5	12.50	7	10	87.86	35.144
6	5	7	65	99.26	99.26
7	20	11	120	7.08	1.77
8	12.50	7	120	77.52	31.008
9	12.50	3	65	9.08	3.632
10	20	3	10	3.16	0.79
11	12.50	7	65	84.68	33.872
12	20	7	65	87.14	21.785
13	12.50	11	65	96.84	38.736
14	5	3	10	6.54	6.54
15	5	3	120	14.54	14.54
16	20	11	10	90.58	22.645
17	5	11	120	97.52	97.52

**Table 2:** BET (Brunauer–Emmett–Teller) surface area, pore-volume, and pore size of different adsorbents.

Materials	BET Surface Area (m^2^/g)	Pore-Volume (m^3^/g)	Pore Size (nm)	References
Hybrid CNTs	71.24	0.230	12	This work
Synthesized CNTs	57.35	0.01	10	[45]
CNT/ZnCo_2_O_4_	67.60	1.103	-	[46]
CNMH	164.60	0.29	9	[47]
CNT/TiO_2_	51.44	0.67	9.33	[48]

**Table 3:** Zeta potential results of hybrid CNTs with different pH values.

Sample	pH	Zeta Potential (MV)
1	3	0.111
2	5	−5.255
3	7	−7.602
4	9	−10.558
5	11	−23

**Table 4:** ANOVA for Al^3+^ removal of hybrid CNTs.

Source	Sum of Squares	df	Mean Square	F Value	P−Value Prob > F
Model	0.27	9	0.030	3.99	0.0409
A-Dose	8.642 × 10^−3^	1	8.642 × 10^−3^	1.14	0.3209
B-pH	0.074	1	0.074	9.81	0.0165
C-Contact Time	9.866 × 10^−3^	1	9.866 × 10^−3^	1.30	0.2913
AB	8.801 × 10^−3^	1	8.801 × 10^−3^	1.16	0.3168
AC	4.389 × 10^−4^	1	4.389 × 10^−4^	0.058	0.8167
BC	0.092	1	0.092	12.16	0.0102
A^2^	2.525 × 10^−4^	1	2.525 × 10^−4^	0.033	0.8603
B^2^	0.040	1	0.040	5.32	0.0544
C^2^	6.937 × 10^−4^	1	6.937E × 10^−4^	0.092	0.7710

**Table 5:** ANOVA for Al^3+^ capacity of hybrid CNTs.

Source	Sum of Squares	df	Mean Square	F Value	P − Value Prob > F
Model	15582.14	9	1731.35	14.14	0.0010
A-Dose	5774.65	1	5774.65	47.17	0.0002
B-pH	4186.28	1	4186.28	34.19	0.0006
C-Contact Time	10.58	1	10.58	0.086	0.7774
AB	3638.90	1	3638.90	29.72	0.0010
AC	11	1	11	0.090	0.7731
BC	292.46	1	292.46	2.39	0.1661
A^2^	1181.14	1	1181.14	9.65	0.0172
B^2^	901.39	1	901.39	7.36	0.0301
C^2^	111.47	1	111.47	0.91	0.3718

**Table 6:** Adsorption kinetics and correlation coefficient.

			Pseudo-First-Order *ln(q_c_ − q_t_) vs time (t)*	Pseudo-Second-Order *(t/q_c_ vs t)*	Intraparticle *(q_c_ vs t^0.5^)*
Dose mg	pH	C_0_ mg/L	R^2^	R^2^	R^2^
13.5	7	3	0.9091	0.9583	0.8769
13.5	7	5	0.7084	0.999	0.522
13.5	7	10	0.8275	0.8736	0.874

**Table 7:** Comparison between the maximum adsorption capacity of hybrid CNTs with other absorbents.

Absorbent	Pollutant	Capacity (mg/g)	References
Hybrid CNTs	Al^3+^	347.88	This study
MWCNT	RhB	568.181	[60]
mesoporous composite γ-Fe_2_O_3_/α-Fe_2_O_3_/CA	RhB	165	[61]
P*n*,*n*-CNTs	MO	263.14	[62]
magnetic-modified multi-walled carbon nanotubes	MB	48.1	[63]
