# Erratum: Synthesis, Characterization, and Analysis of Hybrid Carbon Nanotubes by Chemical Vapor Deposition: Application for Aluminum Removal. *Polymers* 2020, *12*, 1305

**DOI:** 10.3390/polym12081702

**Published:** 2020-07-29

**Authors:** Alfarooq O. Basheer, Marlia M. Hanafiah, Mohammed Abdulhakim Alsaadi, Wan Zuhairi Wan Yaacob, Y. Al-Douri

**Affiliations:** 1 Department for Earth Sciences and Environment, Faculty of Science and Technology, Universiti Kebangsaan Malaysia, Bangi, Selangor 43600, Malaysia; mhmarlia@ukm.edu.my (M.M.H.); yaacobzw@ukm.edu.my (W.Z.W.Y.); 2 Centre for Tropical Climate Change System, Institute of Climate Change, Universiti Kebangsaan Malaysia, Bangi, Selangor 43600, Malaysia; 3 Nanotechnology and Catalysis Research Center (NANOCAT), University of Malaya, Kuala Lumpur 50603, Malaysia; m.hakim@unizwa.edu.om (M.A.A.); yarub@um.edu.my (Y.A.-D.); 4 National Chair of Materials Science and Metallurgy, University of Nizwa, Nizawa 611, Oman; 5 Department of Civil Engineering, Almaref University College, Al-Anbar 31001, Iraq; 6 University Research Center, Cihan University Sulaimaniya, Sulaymaniyah 46002, Iraq; 7 Department of Mechatronics Engineering, Faculty of Engineering and Natural Sciences, Bahcesehir University, Besiktas, Istanbul 34349, Turkey

The authors wish to make a change to the published paper [1]. We wish to add Marlia M. Hanafiah as a coauthor to this paper, and an affiliation of Marlia M. Hanafiah—Centre for Tropical Climate Change System, Institute of Climate Change, Universiti Kebangsaan Malaysia, Bangi 43600, Selangor, Malaysia. The contribution of Marlia M. Hanafiah was supervision and formal analysis, and the revised version of the Author Contributions section is as follows:

**Author Contributions:** Synthesis and laboratory work, A.O.B.; Writing—original draft preparation, A.O.B. and M.A.A.; Supervision, M.M.H. and W.Z.W.Y.; Writing—review and editing, Y.A.-D.; Formal analysis, M.M.H. All authors have read and agreed to the published version of the manuscript.

The authors apologize for any inconvenience caused and the change does not affect the scientific results. The manuscript will be updated, and the original will remain online on the article webpage at https://www.mdpi.com/2073-4360/12/6/1305.
